# The Use of Video Instructions in Patient Education Promoting Correct Technique for Dry Powder Inhalers: An Investigation on Inhaler-Naïve Individuals

**DOI:** 10.3390/pharmacy6040106

**Published:** 2018-09-29

**Authors:** Sofia von Schantz, Nina Katajavuori, Anne M. Juppo

**Affiliations:** 1 Faculty of Pharmacy, Department of Pharmaceutical Chemistry and Technology, University of Helsinki, P.O. Box 56, 00014 Helsinki, Finland; anne.juppo@helsinki.fi; 2 The Centre for University Teaching and Learning (HYPE), University of Helsinki, Siltavuorenpenger 1 A P.O. Box 9, 00014 Helsinki, Finland; nina.katajavuori@helsinki.fi

**Keywords:** dry powder inhaler, inhaler technique, inhaler education, asthma

## Abstract

**Introduction:** The correct use of a prescribed inhaler device is crucial for achieving successful disease management in asthma. This study investigates non-verbal, demonstrational videos as a method of teaching inhaler naïve individuals how to use a dry powder inhaler (DPI). **Methods:** Video instructions for four DPIs were examined using a mixed methodology; 31 inhaler-naïve individuals participated in the study. Participants were each shown a demonstrational video of one the four inhalers, after each video the participant demonstrated how they would use the inhaler. After demonstrating the use, participants crossed over to the next inhaler. The demonstrations were videotaped. A common questionnaire was filled at the beginning of the study and four inhaler-specific questionnaires which were filled out by the participant after each inhaler demonstration. **Results:** The frequency of participant error varied between inhalers. When asked about how they perceived the video instructions, participants often stated they would have liked to receive feedback on their performance. The importance of feedback was further highlighted by the fact that participants tended to overestimate their own inhaler technique. **Conclusion:** Non-verbal videos may be more efficient for some DPIs than for others as a method for providing inhaler instructions. Lack of feedback on the participants’ inhaler performance emerged as a clear shortcoming of this educational method. Some steps in the inhalation process may be harder for individuals to remember and therefore require extra emphasis in order to achieve correct inhaler technique.

## 1. Introduction

Asthma is a chronic respiratory disease that may affect as many as 334 million people worldwide [1]. Asthma has become the most prevalent chronic disease in developed countries and affects over 10% of the adult population [2]. The disease is most often treated with inhaled therapies [3], and today dry powder inhalers (DPIs) represent the most rapid-expanding type of device in the treatment of asthma. [4,5]. As such, the correct use of DPIs is important in order for patients to receive the benefits of a proper treatment.

Patient education plays one of the most important roles in the patients’ use and misuse of asthma inhalers [6]. Incorrect inhaler use is common and the cost implications are many and appear in such areas as additional doctor visits, hospitalizations and increased use of prescribed inhaler medication. Research suggests that costs can be reduced by spending more time on teaching patients how to correctly use their prescribed inhaler device of inhaler use [7,8]. To counteract for the increasing costs, new and efficient ways of enhancing inhaler technique and safe use of asthma inhalers are needed. Current approaches for providing inhaler training include written instructions, illustrations, audiovisual demonstrations, interactive computer programs, as well as personal and small group demonstrations [9,10], but not all forms of instructions are not equal. Roberts et al. [11] suggested that provision of the manufacturer’s instruction sheet alone was ineffective as a method of providing inhaler instructions, partially because patients tended to overlook this information.

Traditionally, healthcare professionals (HCPs) have played an important role in achieving correct inhaler technique and maintaining it over time. Personal instructions provided by a pharmacist have been found more effective than written instructions, and the inclusion of a physical demonstration was found to improve the instructed patient’s inhaler technique [12]. Personal and small group demonstrations by trained professionals can be considered adequate possibilities for providing inhaler education, but these alternatives are also often costly [13,14]. Many HCPs, however, exhibit difficulties when asked to demonstrate the correct technique for asthma inhalers [6,15]. Research suggests that 39–67% of nurses, respiratory therapists and doctors are unable to sufficiently describe, or perform, critical steps for inhaler use [6]. Pharmacists have also been found to exhibit insufficient inhaler technique [15]. This may point to a lack of training provided to these groups. Clinicians’ ability to use inhalers is typically 5–8 years behind the introduction of new devices [16]. When having received training, HCPs such as pharmacists have been found to be well suited to improve inhalation technique [17]. Nevertheless, problems experienced by HCPs points to the usefulness of standardized teaching methods, such as video education. 

Video education and its use among HCPs has become increasingly used in recent years [18]. The videos used in this study are available and used as educational tools in pharmacies across Finland. Video-based education has been used as a tool for providing patients with health education and correct treatment guidelines for individuals with or belonging to risk groups of heart failure and asthma among others [19,20]. Inhaler instructions via video have been suggested as an affordable way to provide easily accessible and standardized inhaler instructions at any time anywhere, and video demonstrations have been found to promote recall on inhaler use in asthma patients [18]. 

Even though several factors indicate the usefulness of video education and video instructions are becoming an increasingly common ways of providing inhaler education, little research regarding the efficiency of this educational method exists. This study aims to evaluate the use of non-verbal, inhaler specific educational videos as a method of providing inhaler education to inhaler naïve participants with the objective of teaching them how use an inhaler correctly. The study also focuses on identifying areas in which video instructions can be developed in order to fit the needs of individuals without prior inhaler experience. 

## 2. Materials and Methods

The data was collected using a mixed methods study design [21]. The four inhalers used in this study were Diskus, Easyhaler, Ellipta and Turbuhaler. Observational and quantitative data was collected through videotaping of the participants’ inhaler performances after having received video education on correct inhaler use. Additionally, qualitative data was collected through semi-structured interviews, after each inhaler performance. All additional comments made about the inhalers and inhalation videos during the data collection process were written down.

### 2.1. Recruitment

Non-asthmatic, inhaler-naïve, individuals within the age range of 25–34 years were recruited from the general public through flyers posted in several public locations, such as libraries, super markets, cafés, universities and vocational schools in the Helsinki-metropolitan area in Finland.

Participants were considered inhaler-naïve if they did not have asthma or any experience working with, using or helping others to use inhalation devices. A prerequisite for participation was that participants had to be entirely inexperienced regarding inhaler use. Participants who had family members who had asthma were also excluded. The requirement of inhaler naivety was to reduce potential bias against any of the inhalers used in the study.

### 2.2. Pilot

Before beginning the data collection, the process and the questionnaires were piloted on one individual. Based on the feedback from this individual, the process was standardized. A process chart for how the conduct data collection process was established and the information that was to be provided to the participants was written down and standardized. During the interview it emerged that the pilot had previous experience of inhalers that was not disclosed prior to the study. In order to reduce potential biases towards any of the inhalers the pilot individual was excluded. Due to difficulty finding participants no further pilot study was conducted.

### 2.3. Study Population and Sample

The primary reason for choosing individuals aged 25–34 was that statistics from the Finnish National Institute for Health and Welfare (THL) suggested that asthma had most frequently been diagnosed in this age group in the adult population in Finland [22]. Additionally, previous research suggests that elderly individuals may find it difficult to operate inhalers, since poor manual dexterity, weakness, and visual limitations present potential problems affecting inhaler use among the elderly [23]. As such, a deliberately young study population was chosen in order to reduce age-related inhalation errors and, as such, focus on the evaluation of video instructions as a method of providing inhaler education.

In total, 31 individuals (excluding the pilot) completed the study. Another two individuals had announced their willingness to participate, but were excluded due to prior inhaler use. The mean age of the participants was 28 years and the distribution of men and women was 17:14 (55%:45%). A clear majority of participants had an academic education (74%, 23/31), another 5 (16%) participants had attended a university for applied sciences and 3 (10%) participants had completed secondary education. All participants lived or worked in the Helsinki Metropolitan Area.

### 2.4. Data Collection

The data was collected over the span of 1.5 months (04/2015–05/2015). Data collection was carried out in quiet rooms at the University of Helsinki and Hanken School of Economics and all data collection sessions were carried out without interruptions. Each participant had been asked to reserve approximately hour for participation. The approximate length of participation time was 45 to 60 min. The length of the videotaped demonstrations varied from person to person (interval 13–53 s and mean: 30 s). The length of the educational videos varied between 1:30 min and 1:50 min. The rest of the time was spent filling in the questionnaires and talking to the participants. 

**Figure 1:**
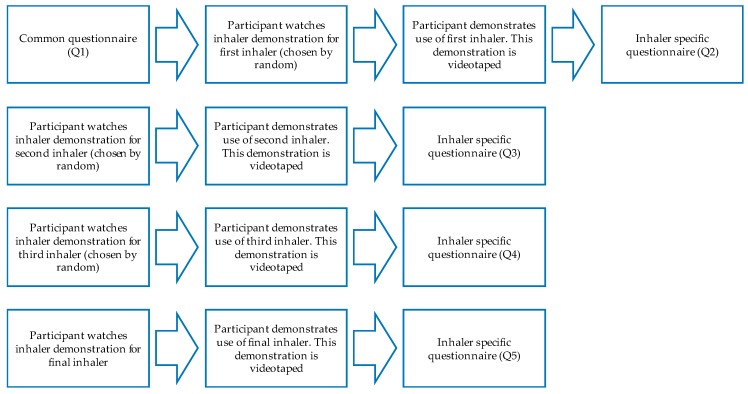
Flow chart depicting the different steps for data collection.

The data gathering process included the participants watching educational videos of four inhalers and demonstrating the use of these inhalers, filling in five questionnaires as well as freely interacting with the participants and taking notes of additional comments made about the inhalers as well as the instructional videos. In an attempt to increase the reliability of the analysis, each participant was given an identification code and their questionnaire responses were linked to their inhaler scores and treated as anonymous. The material analyzed for this study included 155 questionnaires and 124 videotaped inhaler demonstrations. A flow chart of the data collection process is presented in Figure 1.

The questionnaires were developed by the authors based on key areas that were identified after a literature review on inhaler use, inhaler errors and inhaler education. The inhaler-specific questionnaires (Q2–Q5) were identical and contained both open-ended and closed questions. The main purpose of the closed questions was to collect data in which characteristics of the different inhalers and the videos could be easily compared to each other. In the closed questions, participant responses to questions assessing their perception of different aspects of the devices were elicited using a five-point Likert scale (strongly disagree, disagree, neutral, agree and strongly agree). The open questions were designed to give participants an opportunity to freely express their opinions and thoughts on the inhalers, as well as the educational material shown to them. Examples of the open-ended questions from the inhaler specific questionnaires can be observed in Table 1. 

First, the participants filled in a common questionnaire (Q1) with questions regarding demographics, such as gender, educational background, and home town. In addition, the questionnaire contained questions designed to make sure the participants had no prior inhaler experience. After watching the educational video for a specific inhaler, the participants were asked to demonstrate their first attempt at using the inhaler correctly. This demonstration was videotaped. No additional verbal or demonstrative instructions were given. Participants then moved on to the other inhalers in a random order. The order was determined by lottery and drawn before the participant started watching the first demonstrational video. A random order was chosen with the purpose of minimizing the effect that ordered demonstrations would have on the results. The participants’ personal opinions of the inhalers, the video material and self-evaluation of their inhaler performance were assessed using a self-completed questionnaire. Participants’ additional spontaneous comments relating to the inhalers or the video material were written down and used to complement the answers in the open-ended questions. The participants’ inhaler demonstrations were videotaped and analyzed by the researcher both during and after the demonstration, using an inhaler-specific checklist.

### 2.5. Educational Videos

The non-verbal demonstrational videos were produced by the Association of Finnish Pharmacies [24] and developed as a collaboration between the association and representatives from the pharmaceutical companies representing each device. The videos are used as training tools by pharmacies across Finland and are publicly available. The videos build on the information provided in the patient information leaflets (PILs), which have been approved by regulatory authorities. The videos can be divided into four sections that are common for all videos: introduction of the device; inhalation instructions’ description on how to read the dose counter; as well as instructions on how to clean the device. The videos for Diskus and Ellipta also contain information regarding rinsing of the mouth after use. Participants were asked not to demonstrate mouth rinse. 

During the instruction phase, written instructions for every step appeared on the bottom of the video screen. The steps were performed in chronological order and the entire inhalation process was demonstrated. As the written instructions appear, each correct step of the inhalation process is demonstrated by a trained individual. The trained individuals demonstrating the device are different in each video. The phase which describes how to read the dose counter follows the same format. Information regarding the dose counter is written below a still image of the device. The dose counter is highlighted using a red arrow or a red circle. The videos use standardized terminology that can be found in the PIL.

### 2.6. Analysis of Data

#### 2.6.1. Frequency and Characterization of Errors

The videotaped inhaler demonstrations were checked against a predetermined checklist. This was repeated twice by the first author. As a control measure, the second author made random controls. When determining the frequency of error for each inhaler, only errors that could influence the efficacy of treatment were noted. For example, not closing the cap of the inhaler properly was not noted as a critical error. This error could potentially affect the stability of the product long term, but does not directly affect the measured inhalation. As such this error was not noted.

**Figure 2:**
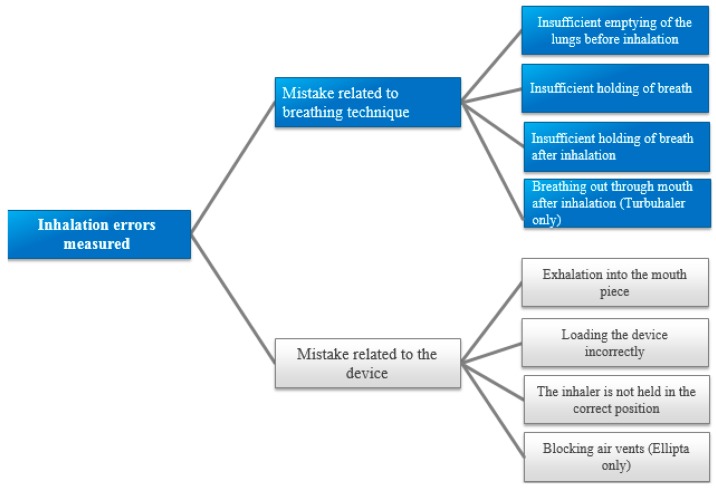
The errors measured throughout the study.

Errors were defined as: displays of flawed technique or lack of knowledge regarding usage of the inhaler device that conflicted with the inhaler instructions provided by the manufacturers or were causing non-optimal inhaler effect. The errors measured from previous studies were used when compiling the checklist [25]. The errors measured can be observed in Figure 2. Table A1 shows the inhaler instructions participants received for each inhaler in the educational videos, as well as the errors deemed critical for each inhaler. The error assessments for each inhaler type vary somewhat depending on the instructions given to participants. For example, ”Holding one’s breath after the inhalation” was assessed for Diskus, Easyhaler and Ellipta, but not for Turbuhaler, because this step was not included in the video instructions. 

In the context of this study a faulty inhaler performance was defined as one where participants made at least one error. A correct inhaler performance was defined as an inhaler performance where the participant made zero critical errors. The self-evaluated correct use was measured by asking the participants whether they believed they had used the inhalers correctly.

#### 2.6.2. Analysis of Semi-Structured Questionnaires

Participant comments and answers to open questions were analyzed using *qualitative content analysis* [26]. First, all comments and answers to questionnaire questions were gathered into one Word document and read several times without applying any specific framework. After this, sentences that were relevant for the objective of this study were separated out and condensed, still bearing in mind the context in which they were said. Thereafter, the condensed units were grouped into categories. Finally, the categories were processed in order to find underlying themes belonging to each of the categories. In the results section, all participants are given an identification number. All quotes were translated from Finnish to English by the authors.

### 2.7. Ethics

Approval for the materials distributed and the methodology used was obtained by the Ethical Review Board in the Humanities, Social and Behavioral Sciences at the University of Helsinki (statement 4/2015). All participants signed consent forms and were informed how the data would be gathered and stored. Participants were informed about the study and informed of who would have access to the data and assured of their anonymity. Participants were told that they could stop the process at any time without consequences. Participants all gave written informed consent to participate in the study, and their data were anonymized.

## 3. Results

### 3.1. Frequency and Characterization of Errors

Inhaler error frequency varied between the four DPIs. Participants’ self-evaluated correct use and actual correct use varied greatly (Table 2) and they tended to overestimate their own inhaler technique. Many participants believed they were exhibiting correct inhaler technique when they, in fact, exhibited at least one inhalation error. As seen in Table 2, this phenomenon could be observed for all four inhalers. 

The participants’ personal perception of which inhalers were easy to use, and which were difficult, seemed to relate to the actual results for correct use. Upon self-evaluation, participants most often perceived that they had used Diskus and Ellipta correctly. These were also the inhalers for which participants exhibited the fewest number of errors during handling. In contrast, Turbuhaler was the inhaler for which participants appeared to be the most unsure about their inhaler technique. This was also the inhaler for which participants exhibited the highest frequency of error. 

**Figure 3:**
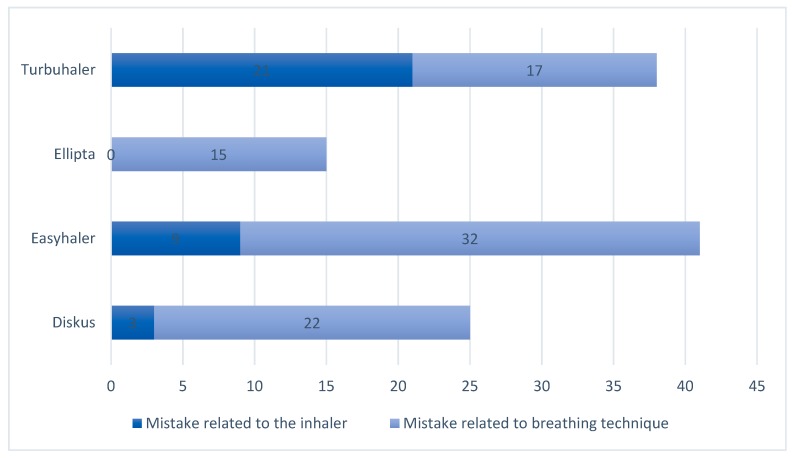
Inhaler-related and breathing-related inhalation errors made by participants.

When comparing the most common inhalation errors, insufficient emptying of the lungs emerged as the most frequently occurring inhalation error for all inhalers. For Turbuhaler, failure to load the device was as common as insufficient emptying of the lungs. The total number of inhaler related and breathing related errors for each device type can be found in Figure 3.

### 3.2. Results from Semi Structured Interviews

Three major categories were identified when analyzing the semi-structured interviews. For the first category, three themes were identified, for the second category another three themes were identified and in the final category six themes were identified. The themes for each category, as well as examples, can be seen in Table 3.

Step-by-step explanations, as well the visual aspect of the educational material, were described as positive and learning-enhancing factors. Factors contributing to a negative perception of a video included comments on the tempo of the videos as well as the medium’s lack of interaction. Recurring feedback from participants for all four videos was that the tempo was too fast. The educational video for Ellipta received the most negative comments with many participants stating that the fast tempo made it difficult to follow the instructions properly. The instructions for the Easyhaler video were also criticized for being difficult to understand and having a fast tempo. Some participants (5/31) also commented that they thought a video was a better way of teaching the use of an inhaler than just providing them with written instructions. Participants mentioned unanswered questions, unclear instructions and the lack of feedback as factors they perceived to be difficult with the video instructions. Uncertainty regarding their own technique was also mentioned as a problem related to the video instructions. The addition of a spoken track, music or close-up captures were suggested as ways of enhancing the function of the video training. 

When asked what kind of inhaler education would need to be provided for them to best understand inhaler instructions, participants stated that the instructions should be clear and show the inhalation process step-by-step, focusing on loading the device as well as proper breathing technique. The videos used in this study all contained the above-mentioned steps. It was also suggested that combining the video education with written instructions, or a checklist of the different steps, might have been a good reminder of how to use the inhaler. Another frequently reoccurring theme was feedback, and participants also commented that they would have liked to receive feedback on their inhaler performance. The general perception was that face-to-face instructions would be good in the beginning to assure that the inhalation is done correctly. One participant explained:

“*I think it would be good if someone checked whether the inhaler is used correctly. After this, the video could work as a great supplement. Overall, I think the video was clear and the tempo was slow enough.*” Participant nr 1 (PN1).

Similar statements occurred among other participants as well. Some participants explicitly stated that they thought videos were a good or adequate way of providing inhaler instructions (6/31). Others disagreed and highlighted that watching an instructional video once was not enough when teaching correct inhaler technique (5/31). This is an interesting observation, since most participants still answered that they believed they had used the inhalers correctly. The lack of feedback and opportunity to ask questions also led individuals to misunderstand the function of the inhalers. These misunderstandings emerged from the comments made during and after the inhalation process. In addition, participants frequently asked questions after the inhalation process or highlighted questions that they wished the videos would have answered. The type of questions and misunderstandings varied greatly among participants. For example, one participant (PN7) stated that he would have liked more information on the dosage and how it could be adjusted on the inhaler. In fact, none of the inhalers provided the option to adjust the dosage for one single inhalation. Another participant highlighted the issue of storing the product, wondering if it mattered where the product was stored (PN18), and others discussed shaking one of the inhalers and wondered why this was necessary (PN7 and PN6).

In each of the videos shown to participants, written instructions for every step appeared on the bottom of the video screen during the inhalation process. In addition to comments regarding the fast tempo of some of the videos, participants also commented that the text flashed by very fast and that it was hard to simultaneously concentrate on reading the text in the video, look at the visual instructions and let the instructions sink in. It was suggested that verbal instructions in the video may be more favorable compared to written, as this would allow the participant to concentrate on the person showing the correct technique instead of concentrating on reading the instructions.

## 4. Discussion and Conclusions

### 4.1. Discussion

The high frequency of error for the four inhalers observed in the results seem to indicate that the non-verbal videos used for this study were an inadequate way of providing inhaler education for first time users of inhalers. The perceived and actual level of difficulty varied between the inhalers, and some inhalers proved to be either harder to use or harder to teach through non-verbal video instructions. (Table 2). As such, one could argue that non-verbal, PIL based educational videos as a method of providing inhaler education may be more efficient for some DPIs than for others. Alternatively, the difference could be explained minor by differences in the instructions provided or differences between the videos. The differences between the videos were consciously minimized by choosing videos with the same format and structure. Based on comments from participants, the pace appeared to be slightly different. 

Video education could potentially be considered a cost-effective way of providing inhaler education, at least for some DPIs. The use of video education is supported by previous research suggesting that patient knowledge and understanding can be improved by combining visual images and words using video technology [27]. A long-term comparison of small group demonstrations and video-demonstrations and found small group demonstrations of an interactive nature to be the slightly more effective alternative for providing inhaler individual education through video [13]. Van der Palen et al. [13] also found that the improvements achieved both through video education and through group education showed a significant increase from the baseline. In a study comparing written, video and personal instructions for metered-dose inhalers, no significant distinction in inhaler techniques found between patients instructed in person or by videotape [18]. Interventions using a combination of educational videos, checklists, leaflets and verbal instructions found that this type of education significantly improved inhaler technique in patients with COPD, additionally it was found to decrease attack frequency and dyspnea, and improved quality of life [28]. There is clearly potential for the development of easily accessible educational methods of providing video instructions, but more work needs to be done to understand how the needs of patients can be met using video instructions.

Inhaler technique has been found to deteriorate over time (potentially after as little as 2–3 months) [29]. As such, inhaler handling training must occur regularly in order to achieve and maintain the correct technique [30,31]. Based on the rather high frequency of participant error in this study, it could be proposed that providing inhaler education via video would be more suitable as a means of promoting recall in current inhaler patients than when teaching individuals how to use an inhaler for the first time. This is supported by Wilson et al. [19] who suggests that the use of video and print interventions can promote recall on inhaler use in asthma patients. A study examining personal demonstrations by pharmacists as a learning tool concluded that at least three repetitions of device instructions were needed in order to achieve errorless technique, or less than 10% errors in total [32]. As such, it would be interesting to investigate how repeated video trainings may have affected the participants’ inhaler technique. Educational videos distributed online allow for the patients to access the videos at any time in order practice their inhaler technique. There may however be a significant threshold for patients to go looking for these videos on their own initiative, as real-world patients have been known to overlook other easily available educational sources such as leaflets [11]. 

An interesting observation in this study was that participants tended to overestimate their own inhaler technique. This was apparent for all four inhalers. This is supported by previous research in patient populations suggesting that patients are often not aware of the fact that they use their inhalers inadequately, and they often overestimate their own abilities [33]. The observation is problematic given the concept of video instructions, since the participants are not able to receive feedback on their inhaler technique and may therefore continue making mistakes. The results suggested that the participants would have needed feedback on their own performance, and personal demonstrations with an HCP was often suggested. Correct face-to-face training with feedback supports the patients’ treatment by making them aware of their inhalation errors, thereby providing clear guidelines on required technique. The high costs of these types of sessions, however, limit their use in real-life situations and new alternatives are still needed to assure easy and cost-efficient access to inhaler education. Despite the participants’ apparent preference for face-to-face demonstrations, many questions regarding this still remain open, as forms of video education have been found to be equal or superior to personal inhaler demonstrations [13,18]. Another argument presenting the potentially problematic side of real world one on one training is that HCPs such as doctors, nurses, respiratory therapists and pharmacists have been shown to exhibited difficulties when asked to demonstrate the correct technique for inhalers [6,15]. As such, it is important to emphasize, that in order for one on one training to be efficient the HPC must have sufficient understanding and training of the use of these devices.

#### 4.1.1. Potential Areas for Development in Educational Videos for Asthma

When developing video instructions and educational material instructing on the use of inhalers, focusing on developing means for providing feedback and interaction would be essential. Results indicated that participants were often left with unanswered questions regarding the inhalers after watching the video instructions. Clear misconceptions about information provided in the videos also emerged. These varied greatly between individuals, which was interesting given that all participants had received identical instructions. Still, they seemed to interpret and focus on different parts of the information. In order for some inhaler-naïve individuals to achieve correct technique, it would be important to provide mechanisms for feedback and correcting misconceptions and flawed inhaler technique.

One option would be developing interactive videos or mobile applications, since educational research appears to suggest that interactive educational videos achieved significantly better learning performance than non-interactive videos [34]. The importance of providing feedback could also be incorporated into the inhaler devices themselves in the future. Furthermore, interactive chats with HCPs could be considered a way of allowing individuals to ask questions and clear up misunderstandings that arise while learning inhaler technique through video. The combination of video and written instructions was also suggested by some of the participants in this study. Orion Pharma has recently included a QR code in the updated patient leaflets for Easyhaler, from which patients can scan and access video instructions directly on their phones. This represents an interesting and innovative way of combining written and visual instructions, as well as making these instructions easily available for the patients [35].

#### 4.1.2. Limitations to This Study

The first limitation of this study was the fact that respiratory flow rate was not measured amongst participants. Slow inhalation has been described as an inhaler mishandling in several studies [36,37]. Since the participants of this study were non-asthmatic, the assumption is that they have inspiratory flow rates typical for a healthy individual. As such their results would not be comparable to those of asthma patients if these measurements had been made. 

Secondly, it is important to note that the participants in this study were non-asthmatic and inhaler naïve. Although this was an attempt to eliminate a bias towards any specific inhaler, it is important to note that as these individuals did not need the inhalers and as such, may have been less motivation to learn their use them. This may ultimately have impacted their inhaler technique. 

Additionally, the videos used for this study were videos already in use as educational tools for pharmacists and patients alike. While this allowed for a situation closely simulating the current real world scenario of already available videos it was also accompanied by problems. The videos used in this study were all non-verbal, non-interactive of nature. As such the results cannot be extrapolated to all education videos in asthma.

### 4.2. Conclusions

Since a majority of the participant inhaler demonstrations exhibited incorrect inhaler technique, it could be argued that these non-verbal non-interactive video instructions alone are not enough when teaching technique to inhaler-naïve individuals. The most prominent problem with video instructions is the lack of feedback to the user regarding their inhaler performance. This may present a genuine problem, since the results of this study indicated that people tend to overestimate their own technique. 

Breathing technique, especially failure to empty the lungs before the inhalation, was the most common inhalation error among participants. Since the most commonly exhibited error was the same for all four inhalers, it may be fair to conclude that some steps in the inhalation process may be harder for individuals to remember and therefore require extra emphasis in order to achieve correct inhaler technique, especially when teaching individuals how to use an inhaler for the first time. The results may also be indicating that there is considerable room for improvement in video instructions for the purpose of teaching individuals how to use a DPI. Another problem with video education arises from the fact that the users are unable to ask questions and clear up misunderstandings, as expressed by the participants of this study. E-learning and video education could play a part in providing successful inhaler instructions in the future, but if so, more interactive approaches providing patients with the ability to ask questions and receive feedback should be developed. 

### 4.3. Practical Implications

The video instructions in this study were basic, non-interactive, instructional videos that described each step that should be performed during inhalation. Research on instructional videos in e-learning has established that students provided with interactive educational videos achieved significantly better learning performance and a higher level of learner satisfaction than those who were provided with a non-interactive video, or no video at all [34]. These findings indicated that it may be important to integrate interactive instructional video into e-learning systems. If video instructions were to be more widely used as a method of providing instructions on inhaler technique for inhaler-naïve individuals, it would be interesting to consider the possibility of developing interactive videos. Future research should contemplate whether these types of videos would be suitable for providing inhaler education to patients, and how different types of interactive videos could assist in the goal of achieving and maintaining correct inhaler technique. This study was performed on a young population, an interesting subject for future research would be to investigate this phenomenon on an older population. Investigation on how repeated video training affects inhaler technique would also be of interest. Furthermore, instead of looking at video as a tool for providing inhaler education to inhaler-naïve individuals, future research could investigate video education as an educational tool for maintaining correct inhaler technique in asthma patients on a long-term basis.

## Figures and Tables

**Table 1:** Examples of open ended questions in questionnaires (translated from Finnish).

Example of Ended Questions in Q2–Q5
What kind of instructions would you need on inhaler technique on order to learn how to use the inhaler? How did you feel about the ease of use of the inhaler? Did you learn how to use the inhaler based on the video instructions you received? ◦ Please motivate why? Please motivate why not.

**Table 2:** The self-evaluated correct use and actual correct use for each inhaler type.

	Diskus	Easyhaler ^1^	Ellipta	Turbuhaler
Number of participants who believed they had used inhaler without making a single inhalation error	84% 26/31	77% 24/31	84% 26/31	61% 19/31
Number of participants who actually used the inhaler without making a single inhalation error	48% 15/31	19% 6/31	55% 16/31	16% 5/31

^1^ After the completion of the data collection for this study, the inhaler instructions for Easyhaler were updated by Orion. The update was independent from this study.

**Table 3:** Three major categories emerged in the semi structured interviews. This table shows the categories and subcategories identified and an explanatory quote from each sub-category.

Category	Sub-Category	Explanatory Quote
	Visual instructions	*“I like visual material, so videos worked well for me. This video was short, concise and easy to understand.” PN9*
Factors enhancing performance	Step-by-step instructions	*“In order to learn, I would need clear instructions of each step of the inhaler use. This video was enough.” PN2*
	Type of inhaler	*“The video was good and the inhaler was fairly easy to use. I don’t think you need any other instructions (than the video).” PN31*
	Spoken track	*“I think the video was good and the tempo was fast enough. I think spoken instructions would have been a good addition to the video instructions.” PN21*
Ideas on improving video education	Close-up pictures	*“I think the video could have been improved by adding close-up pictures of the stages, a spoken track or background music.” PN27*
	Complementary material	*“A checklist on the steps to go with the inhaler would probably be good.” PN18*
	Uncertainty of own technique	*“I do think that I used the inhaler correctly but I still feel a little uncertain. I think I would feel better if I could check my technique.”* PN10 *“I do think that I used the inhaler correctly but I still feel a little uncertain. I think I would feel better if I could check my technique.”*
	Unanswered questions	*“I would have liked to know why I had to shake this inhaler but not the others. PN7*
	Inhaler	*“I don’t think I learned how to use this inhaler, at least not very well. The video was good, but using the inhaler was too hard.” PN22*
Factors that were perceived as difficult	Unclear instructions	*“The video started out simple but became confusing as it progressed. I don’t remember what should be done and in which order.” PN28*
	Lack of feedback	*“I think video instructions are much better than text instructions alone. Still, I would like to receive feedback from a pharmacist or someone else who knows how to use it.” PN20*
	Tempo of videos	*“The tempo of the videos was quite fast and I did not have time to read all of the instructions on the screen.” PN19*

## Appendix A

**Table A1:** The inhaler instructions patients received for each inhaler in the educational videos, and the errors deemed critical for each inhaler.

Inhaler	Instructions	Errors measured
Diskus	Open your Diskus: Hold it in the palm of your hand, put the thumb of your other hand on the thumb grip and push the thumb grip until it "clicks" into place. Load the Diskus by holding the device with the mouthpiece toward you. Slide the lever away from you as far as it will go to get your medication ready. Adjust your posture. Keep your shoulders down and head held high. Breathe out away from the device for as long as you feel comfortable. Place the mouthpiece gently in your mouth and close your lips around it. Breathe in deeply and evenly though the Diskus. Remove the device from your mouth. Hold your breath for 5-10 seconds. Calmly breath out through your nose. Close the Diskus by sliding the thumb grip toward you until you hear a “click.”	Failure to open device Failure to hold device in right position Failure to empty lungs before inhalation Failure to load device Failure to hold breath at least 5-10 seconds after inhalation Exhalation into the mouthpiece
Easy-haler	Open the protective cover. Insert the inhaler in a protective case. Make sure that the cover is on the mouthpiece. Remove the mouthpiece cover. Shake the inhaler vigorously up and down 3-5 times. Do not trigger the inhaler when shaking it! Hold the inhaler upright. Trigger the inhaler once until you hear a “click” and let the inhaler return to its original position. Keep holding the inhaler in an upright position. Breathe out normally. Place the mouthpiece in your mouth between your teeth and close your lips tightly around the mouthpiece. Breathe in through your mouth forcefully and deeply. Take the mouthpiece out of your mouth. Hold your breath for at least 5 seconds and then breathe out through your nose. Close the protective cover.	Failure to open device Failure to hold device in right position Failure to empty lungs before inhalation Failure to load device - Failure to shake device - Failure to press button - Loading in wrong order - Triggering inhaler when shaking it Failure to hold breath at least 5 seconds after inhalation Exhalation into the mouthpiece
Ellipta	1. Wait to open the cover until you are ready to take your dose. Do not shake the inhaler. 2. Slide the cover down to expose the mouthpiece. You should hear a “click.” 3. Breathe out away from the device for as long as you feel comfortable. Hold the inhaler away from your mouth - do not breathe out into the mouthpiece. 4. Put the mouthpiece between your lips, and close your lips firmly around it. Do not block the air vent with your fingers. 5. Take one long, steady, deep breath in through your mouth. 6. Hold your breath for at least 3-4 seconds. 7. Remove the inhaler from your mouth. Breathe out slowly and gently. 13. Slide the cover upward as far as it will go, to cover the mouthpiece.	1. Failure to open/load device 2. Failure to hold device in right position 3. Failure to empty lungs before inhalation 4. Failure to hold breath at least 3-4 seconds after inhalation 5. Holding fingers on air vents 7. Exhalation into the mouthpiece
Turbu-haler	Unscrew the cap and take it off. Hold the inhaler upright. Twist the grip of your Turbuhaler as far as it will go. Then twist it all the way back. When twisting you will hear a "click." Breathe out deeply away from the device. Put the mouthpiece between your teeth, and close your lips around it. Breathe in forcefully and deeply through your mouth. Remove the Turbuhaler from your mouth and calmly breathe out through the nose. Replace the cap.	Failure to open device Failure to hold device in right position Failure to empty lungs before inhalation Failure to load device Failure to breathe out through the nose after inhalation Exhalation into the mouthpiece

Source: [13,25,36,38,39,40].
